# Increased intra-subject variability in reward behavior relates to symptom severity in schizophrenia

**DOI:** 10.1038/s41537-025-00645-7

**Published:** 2025-08-05

**Authors:** I-Fei Chen, Yu-Chen Chan, Chih-Min Liu, Yi-Ting Lin, Ming H. Hsieh, Tzung-Jeng Hwang, Tai-Li Chou, Chen-Chung Liu, Yi-Ling Chien, Georg Northoff

**Affiliations:** 1 School of Psychology, University of Ottawa, Ottawa, ON Canada; 2 Mind, Brain Imaging and Neuroethics Unit, Institute of Mental Health Research, Royal Ottawa Mental Health Centre, University of Ottawa, Ottawa, ON Canada; 3 Institute of Learning Sciences and Technologies, National Tsing Hua University, Hsinchu, Taiwan; 4 Department of Psychiatry, College of Medicine and National Taiwan University Hospital, National Taiwan University, Taipei, Taiwan; 5 Department of Psychology, National Taiwan University, Taipei, Taiwan

**Keywords:** Human behaviour, Schizophrenia

## Abstract

Schizophrenia (SZ) is a complex disorder characterized by positive and negative symptoms that have been linked to dysfunction in cognition and reward motivation. Recent findings show higher inter-subject variability in SZ in various cognitive functions. This raises the question of whether there is also higher intra-subject variability in SZ at the psychological level, specifically increased variability across the trials of a psychological task within the subject itself, that is, intra-subject variability. To examine fluctuations in behavior during a reward-based discrimination and liking task, we analyzed intra-subject variability in SZ and observed the following: (i) increased intra-subjective variability across all four behavioral measures, that is, response times (RT) for discrimination and liking tasks, as well as accuracy (ACC) and liking ratings; (ii) significant correlation of the different measures’ intra-subject variabilities across the distinct tasks, e.g., RT, ACC, and liking ratings among each other; and (iii) relation of the increased intra-subjective variability in the behavioral measures (RT, ACC, liking) with overall and general psychopathological symptom severity, as measured by the positive and negative syndrome scale (PANSS). Together, we demonstrate abnormally increased intra-subjective variability in a reward-motivation task in SZ and its key role in relation to symptom severity. This increased intra-subject variability at the psychological-behavioral level suggests abnormal and imprecise timing in cognitive processing, which aligns with analogous findings of temporal imprecision at the neural level.

## Introduction

Variability refers to fluctuations in cognitive performance or behavioral responses. Once considered mere noise, recent studies have emphasized the key role of variability in behavioral performance; for instance, intra-subject variability refers to the degree to which a single subject’s behavioral or cognitive performance changes or fluctuates over time (and trials) in response to one and the same stimulus or task^1–4^. Measures of intra-subject variability, such as subjects’ fluctuations in response time (RT), provide important insights into their cognitive stability or consistency^3–8^. Intra-subject variability can be calculated as coefficient of variation (CV), which divides the standard deviation (SD) by mean RT from trial-by-trial RT of each subject^4,9,10^. Intra-subject variability differs from the mean value: while the mean reflects the central tendency by averaging performance across all trials, intra-subject variability captures trial-to-trial fluctuations and reflect the variation in an individual’s performance, which are typically lost when computing only the mean^6,9,10^. Increased intra-subject variability may indicate cognitive instability, which is associated with poor behavioral performance and fluctuations across tasks such as attention, memory, and executive functioning^6,10,11^.

These intra-subject fluctuations provide valuable insights into the consistency or instability of cognitive processing, particularly in clinical populations such as individuals with schizophrenia (SZ). SZ is characterized by cognitive impairments, featured by increased intra-subject variability in these subjects’ cognition and behavior^3,4,7,8,12^. Studies investigating intra-subject variability in RT during cognitive tasks have found that schizophrenia patients exhibit increased intra-subject variability compared to healthy controls^2,4,13^. This heightened intra-subject variability is thought to reflect an underlying temporal instability in the schizophrenia subjects’ cognitive control mechanisms^4,8^, such that they process cognitive information in a temporally unstable (rather than stable) way. While such variability has been linked to cognitive domains like attention and working memory, it remains unclear whether similar temporal instability is also present in affective functions such as reward processing, and how it may relate to psychopathological symptoms.

Intra-subject variability may serve as a potential biomarker. A recent study by Fan et al. ^14^ introduced a dynamic method analyzing trial-by-trial variability in behavioral data during visual motion perception, allowing a fine-grained examination of intra-subject variability at the psychological-behavioral level. They applied unsupervised clustering (PCA followed by K-means + +) on the fluctuations of the visually perceived stimulus durations (SD) across the trials to identify subgroups among schizophrenia patients. Following clustering, the groups were compared in their SD values, revealing distinct high- and low-irregularity subgroups (HSZ and LSZ). This method enabled characterization of fluctuations in visual perception reflecting its dynamic variability patterns over time. Importantly, their results suggest that the two subgroups may relate to distinct psychopathological symptoms: in the HSZ subgroup, negative symptoms were mediated by the abnormally high visual perceptual variability (SD), whereas in the LSZ subgroup, the low visual perceptual variability indices were linked to positive symptoms. These findings motivate the question of whether analogous increases in intra-subject variability are present in SZ also in other psychological domains beyond sensory perception, such as affective-motivational or reward-related functions; that is the focus of the present study.

The goal of our study is to investigate the intra-subject trial-to-trial variability in SZ during a reward-related behavior as probed by the Monetary Incentive Delay (MID)^15^ (in a modified version without presetting the task difficulty). The rationale behind choosing this task is that SZ is known to exhibit alterations in reward-related activity on the neural level^16–19^ which are related to symptom severity^16,17,19^. Given these findings, we ask the question of whether there are alterations in the intra-subject variability of the behavioral responses in SZ during a reward-related task like the MID.

The first specific aim is to investigate means and intra-subject variability (as trial-to-trial variability) in behavioral responses, specifically response time, accuracy, and liking ratings during the MID task in SZ. Based on the findings of increased intra-subjective variability at the sensory-behavioral level^14,20,21^, we hypothesized increased intra-subject variability in RT, accuracy (ACC) and liking ratings in SZ (compared to healthy subjects) during our MID task. The second specific aim is to correlate the different behavioral measures (RT, ACC, liking) among each other. Given that we assume the increased intra-subject variability to reflect a basic disturbance operating across different domains and functions^21^, we assumed that they would corelate with each other thus reflecting a basic temporal instability across the distinct tasks. The third specific aim is to link such intra-subject variability on the behavioral level to symptom severity. Given that psychopathological symptoms in SZ are related to changes in the reward system^16,17,19^, we hypothesized that the supposedly high intra-subject variability in response time, ACC, and liking ratings relates to psychopathological symptom severity as measured by the PANSS: the higher the intra-subject variability at the behavioral level, the higher the symptom severity.

## Results

### Response time and accuracy in the discrimination task

#### Mean response time and accuracy

**Fig. 1:**
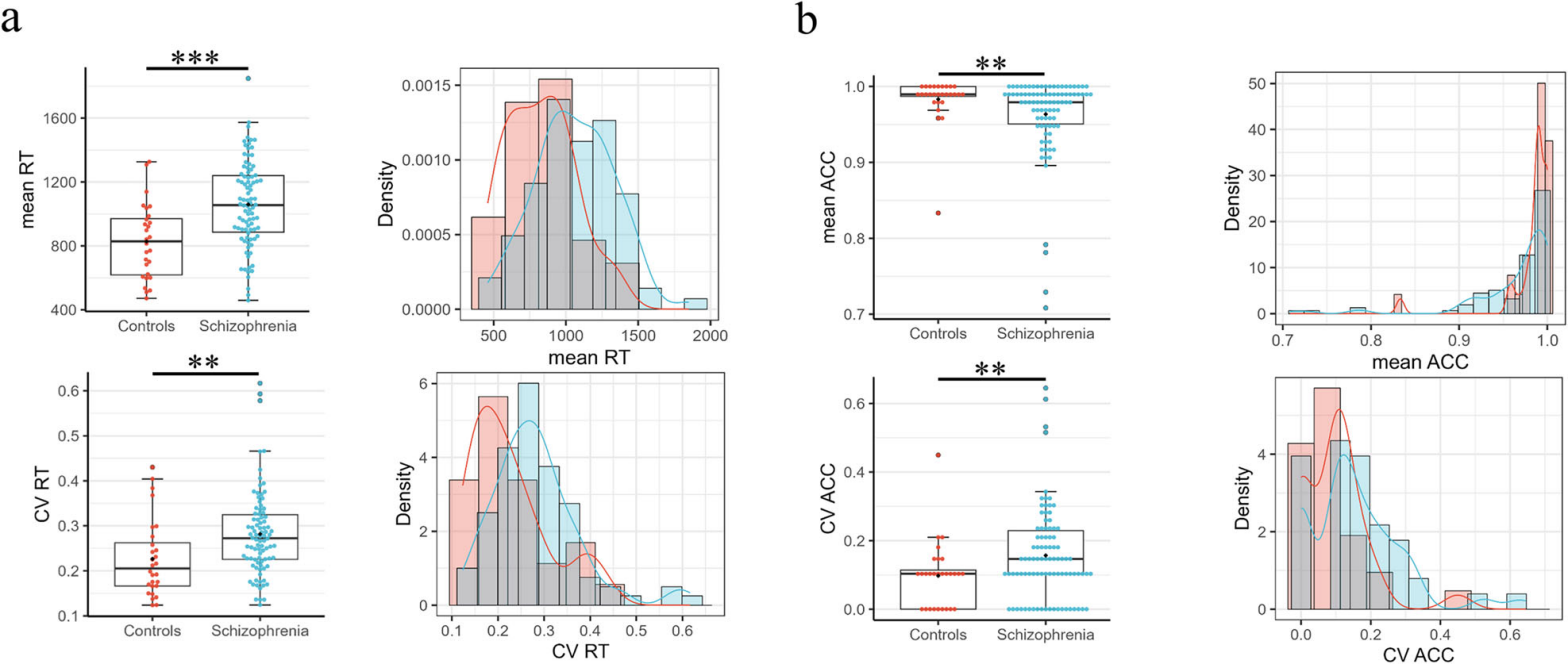
Group differences between schizophrenia patients and healthy controls in the discrimination task. **a** Group differences in the mean and CV of response time across all conditions. **b** Group differences in the mean and CV of accuracy across all conditions. Education was included as covariates. *P < 0.05, **P < 0.01, ***P < 0.001. All statistical analyses were conducted using one-tailed tests.

To investigate non-task-specific group differences, we first analyzed the data without the reward type factor. Since the RT was normally distributed and there was no violation of homogeneity of covariance, the ANCOVA revealed that SZ participants had a significantly higher mean RT across all conditions (*F* (1, 115) = 13.88, *p* < 0.001, η_p_^2^ = 0.100) (Fig. 1a). Next, we included the reward type factor in a mixed factorial ANOVA, and the results again indicated a significant group effect (*F* (1, 115) = 13.88, *p* < 0.001, η_p_^2^ = .108), with SZ participants showing a higher mean RT than healthy controls. There was no significant main effect of reward type (*F* (2, 230) = 2.86, *p* = 0.059, η_p_^2^ = 0.024), and no significant interaction effect between group and reward type for response time was found (*F* (2, 230) = 0.07, *p* = 0.929, η_p_^2^ = 0.0006). Overall, patients with schizophrenia showed higher mean RTs across all conditions thus exhibiting a task-unspecific effect.

In terms of accuracy, Quade’s test for non-parametric ANCOVA was used due to non-normality. SZ participants had a lower mean accuracy across all conditions compared to healthy controls (*F* (1,116) = 4.90, *p* = 0.028) (Fig. 1b). When the reward type factor was included, the results of a robust mixed factorial ANOVA revealed a significant group effect (Q (1, 54.72) = 4.63, *p* = 0.036), but no significant effect of reward type (Q (2, 39.38) = 1.68, *p* = 0.200), or interaction between group (Q (2, 39.71) = 1.41, *p* = 0.256) on accuracy.

#### Intra-subjective variability—increased CV in response time and accuracy

For the CV of the response time across all conditions, Quade’s test for non-parametric ANCOVA showed that SZ participants had a higher CV of RT compared to healthy controls (*F* (1,116) = 8.46, *p* = 0.002) (Fig. 1a). A robust mixed factorial ANOVA showed a significant group effect (Q (1, 31.76) = 14.82, *p* < 0.001), with SZ participants exhibiting higher CV of RT. There were no significant effects of reward type (Q (2, 27.90) = 2.26, *p* = 0.124) or interaction between group and reward type (Q (2, 27.90) = 0.03, *p* = 0.971). Overall, SZ patients showed higher CV of RT across all conditions thus again, as in the mean, exhibiting a task-unspecific effect.

For accuracy, SZ participants also had a higher CV across all conditions compared to healthy controls (*F* (1,116) = 4.90, *p* = 0.028) (Fig. 1b). Including the reward type factor, the results of a robust mixed factorial ANOVA showed a significant group effect (Q (1, 55.28) = 4.16, *p* = 0.046), but no significant effects of reward type (Q (2, 41.43) = 1.61, *p* = 0.212) or interaction between group and reward type (Q (2, 41.53) = 1.56, *p* = 0.223). Overall, SZ patients demonstrated higher CV of accuracy.

### Response time and liking ratings

#### Mean response time and Liking Ratings

**Fig. 2:**
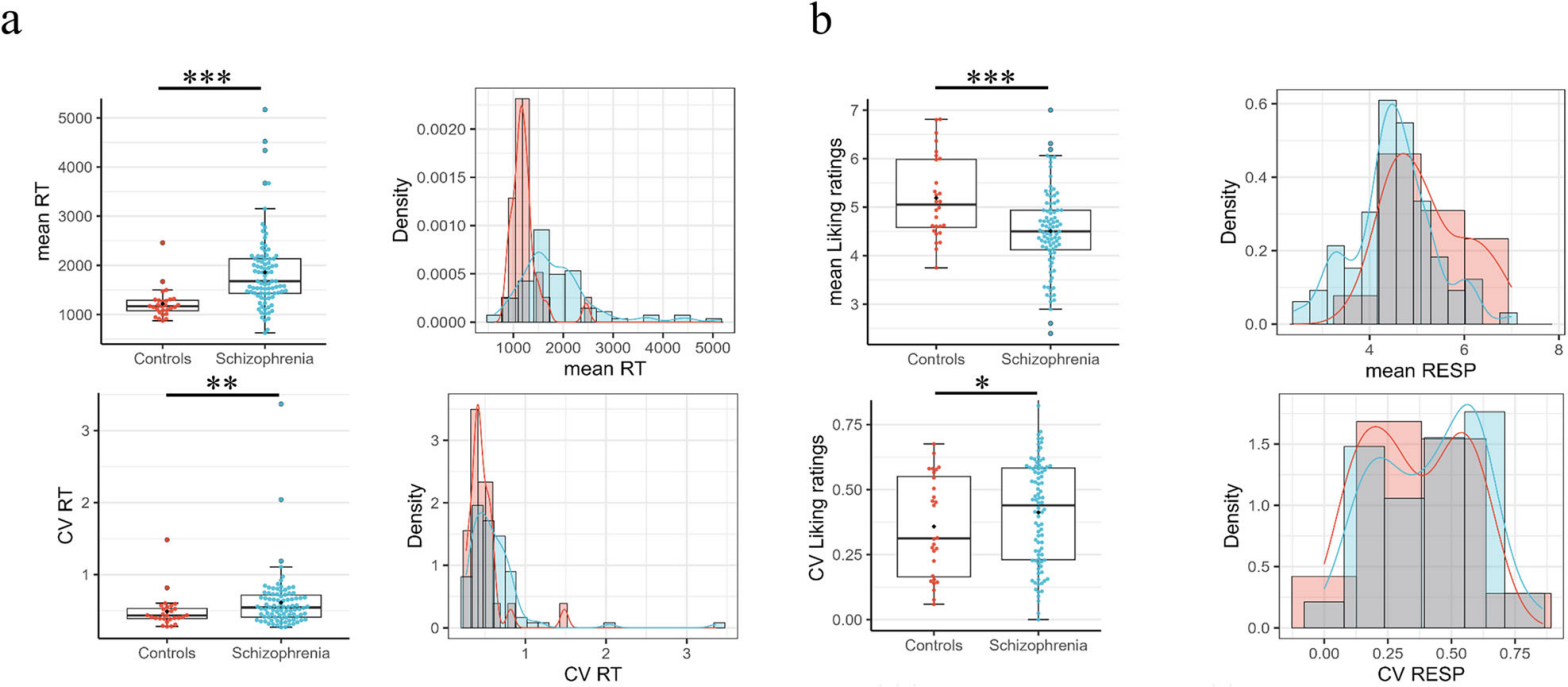
Group differences between schizophrenia patients and healthy controls in the liking rating. **a** Group differences in the mean and CV of response time across all condition. **b** Group differences in the mean and CV of the liking ratings across all conditions. Education was included as covariates. *P < 0.05, **P < 0.01, ***P < 0.001. All statistical analyses were conducted using one-tailed tests.

Due to non-normality in the data, Quade’s test for non-parametric ANCOVA was used to analyze response times in liking ratings. Across all conditions, SZ participants had significantly higher mean response times compared to healthy controls (*F* (1,116) = 31.55, *p* < 0.001) (Fig. 2a). A robust mixed factorial ANOVA revealed a significant group effect (Q (1, 51.86) = 18.60, *p* < 0.001), but no significant effects of reward type (Q (2, 37.72) = 1.99, *p* = 0.151) or interaction between group and reward type (Q (2, 39.20) = 0.02, *p* = 0.985). This shows again task-unspecific effects in the RT mean of the Liking task as in the Discrimination task.

For the liking, SZ participants had a significantly lower mean liking ratings across all conditions compared to healthy controls (*F* (1,116) = 14.69, *p* < 0.001) (Fig. 2b). A robust mixed factorial ANOVA found significant group effects (Q (1, 26.84) = 5.80, *p* = .023) and reward type effects (Q (2, 34.14) = 75.19, *p* < 0.001), but no interaction between group and reward type (Q (2, 32.76) = 0.42, *p* = 0.659). Overall, SZ participants showed significantly lower liking ratings, exhibiting task-unspecific effects as in the accuracy of the discrimination task.

#### Intra-subjective variability—CV response time and liking ratings

SZ participants had significantly higher CV in the response times across all conditions compared to healthy controls (*F* (1,116) = 5.72, *p* = 0.018) (Fig. 2a). A robust mixed factorial ANOVA showed a significant group effect (Q (1, 81.18) = 7.05, *p* = 0.010), but no significant effects of reward type (Q (2, 48.23) = 0.52, *p* = 0.595) or interaction between group and reward type (Q (2, 48.63) = 0.07, *p* = 0.937). Overall, patients with schizophrenia showed higher CV in the RT of the liking ratings in a task-unspecific way.

For the liking ratings, no significant group differences were observed in the CV across all conditions (*F* (1,116) = 1.25, *p* = 0.265) (Fig. 2b). Including reward type factor, the robust mixed factorial ANOVA showed significant effects of group (Q (1, 29.55) = 6.32, *p* = 0.017) and reward type (Q (2, 30.81) = 9.46, *p* < 0.001), with no interaction (Q (2, 31.67) = 0.18, *p* = 0.836).

### Relationship of the increased intra-subjective variabilities among the distinct behavioral measures

We next calculated the correlations between each behavioral measure. The results showed that, for SZ, the variability in RT from the discrimination task is significantly correlated with the variability in ACC from the discrimination task (*ρ* = 0.58, *p* < 0.001) and the variability in RT from the liking ratings (*ρ* = 0.48, *p* < 0.001). Additionally, the variability in ACC from the discrimination task is significantly correlated with the variability in RT from the liking ratings (*ρ* = 0.26, *p* = 0.01). Finally, the variability in liking ratings is significantly correlated with the variability in RT from the liking ratings (*ρ* = 0.23, *p* = 0.03). Together, our findings show significant relationship among the different behavioral measures, e.g., RT, ACC, and liking in their intra-subject variabilities. Although tentative, this pattern suggests that increased intra-subject variability across different behavioral measures may reflect a shared, task-unspecific temporal instability in schizophrenia.

### Relationship of the increased intra-subjective variabilities of the distinct behavioral measures with symptom severity

We then calculated the relationship of the mean of our behavioral variables with psychopathological symptoms; this did not yield major results (see Supplementary Table S1, S2). We therefore raised the question whether variability in our behavioral measures relates to psychopathological symptoms as it was observed in a recent study^4^.

For the discrimination task, intra-subject variability in RT was significantly correlated with PANSS total (*ρ* = 0.26, *p* = 0.01), PANSS general (*ρ* = 0.23, *p* = 0.03): the higher RT variability, the higher the symptom severity in these dimensions. In contrast, no significant correlations were observed with the SANS total or global (*p* > 0.13). Intra-subject Variability in ACC was significantly related to PANSS total (*ρ* = 0.36, *p* < 0.001), PANSS general (*ρ* = 0.37, *p* < 0.001), SANS total (*ρ* = 0.27, *p* = 0.01), and SANS global (*ρ* = 0.27, *p* = 0.01). In general, higher CV RT and ACC in SZ patients were strongly positively correlated with symptoms.

**Fig. 3:**
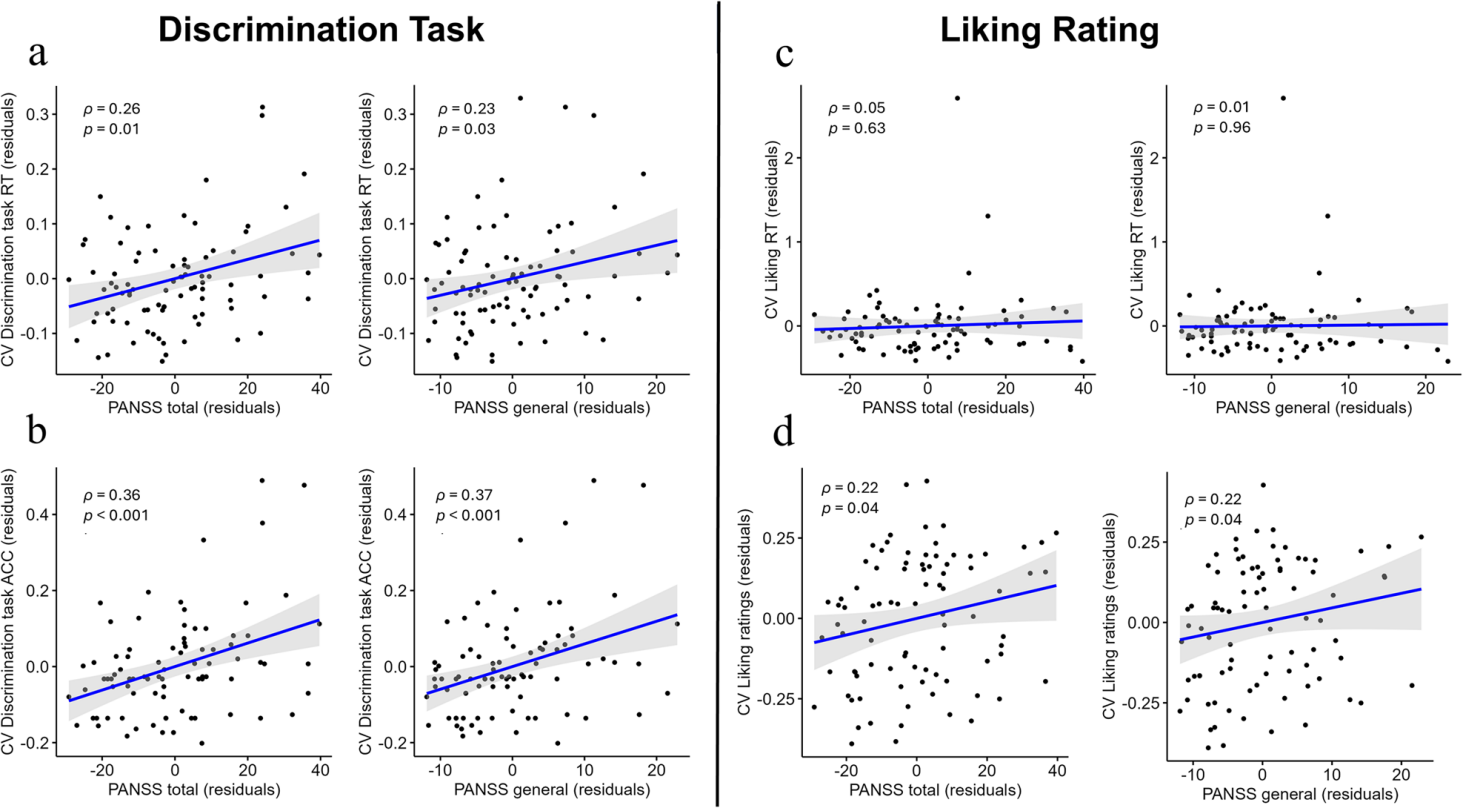
Partial correlation between intra-subject variability and symptoms in discrimination task and liking ratings. **a** The correlation between intra-subject variability in response time during the discrimination task and PANSS total and general scores. **b** The correlation between intra-subject variability of accuracy and PANSS total and general in discrimination task. **c** The correlation between intra-subject variability of response time and PANSS total and general in liking. **d** The correlation between intra-subject variability of liking ratings and PANSS total and general in liking. The partial plots control for education. CV coefficient of variation, RT response time, ACC accuracy.

For the Liking rating, intra-subject variability in response time did not show significant relationships with the PANSS total, general, SANS total, or global scores (*p* > 0.37). Intra-subject variability in liking ratings was significantly related to PANSS total (*ρ* = 0.22, *p* = 0.04) and PANSS general (*ρ* = 0.22, *p* = 0.04). No significant relationships were observed between variability of liking ratings and the SANS total or global scores (*p* > 0.25). Figure 3a–d illustrates the relationship between variability and PANSS total and general scores.

Together, we show robust correlation of the higher intra-subject variability in all four behavioral measures, e.g., RT and ACC of the discrimination task as well as RT and ratings in the liking, with total and general psychopathological symptom severity.

### Effect of medication on mean and intra-subject variability

Given that our subjects were all medicated, there is potential for medication effects. To account for this, we used ANCOVA for parametric statistical analysis and Quade’s test for non-parametric ANCOVA, with CPZ dose as a covariate in the analysis of both mean and CV.

Our analysis revealed no significant group differences in RT intra-subject variability (*F* (1, 116) = 0.13, *p* = 0.72) or ACC (*F* (1, 116) = 0.36, *p* = 0.55) in the discrimination task, nor in RT (*F* (1, 116) = 0.36, *p* = 0.55) or ratings (*F* (1, 116) = 0.01, *p* = 0.94) in liking across all conditions. Similarly, no significant group differences were found in the CV of RT and ACC in the discrimination task for monetary (RT: *F* (1, 116) = 0.06, *p* = 0.80; ACC: *F* (1, 116) = 0.22, *p* = 0.64), humor (RT: *F* (1, 116) = 0.11, *p* = 0.75; ACC: *F* (1, 116) = 0.68, *p* = 0.41), and non-reward (RT: *F* (1, 116) = 0.23, *p* = 0.63; ACC: *F* (1, 116) = 0.04, *p* = 0.84) conditions. Overall, there was significant effect of the CPZ dosage on the intra-subjective variability in the four behavioral measures across all conditions.

This stands in contrast to the medication effects on the mean of the behavioral measures. For mean RT and ACC in the discrimination task across all conditions, a significant group effect was observed for RT (*F* (1, 112) = 10.06, *p* = 0.002) but not for ACC (*F* (1, 116) = 0.36, *p* = 0.55). In the liking, no group effect was found for RT (*F* (1, 116) = 1.59, *p* = 0.21), but a significant effect was seen for ratings (*F* (1, 112) = 9.38, *p* = 0.002). A significant group effect for mean RT in the discrimination task was observed in the monetary (*F* (1, 112) = 8.85, *p* = 0.004), humor (*F* (1, 112) = 9.78, *p* = 0.002), and non-reward (*F* (1, 112) = 10.54, *p* = 0.002) conditions, though no such effect was observed for ACC in any condition. In terms of mean liking ratings, significant group effect was found in the monetary (*F* (1, 116) = 4.84, *p* = 0.003) conditions, but not in the humor (*F* (1, 116) = 2.34, *p* = 0.13) and non-reward condition (*F* (1, 116) = 0.03, *p* = 0.86). No group effect was found for mean RT of liking ratings in any condition. In summary, after accounting for CPZ dose as a covariate, SZ participants demonstrated task-unspecific longer RT mean in the discrimination task and lower liking ratings for monetary rewards. Thus, medication showed a more consistent effect on intra-subject variability than on the mean of the behavioral measures.

## Discussion

The current study investigated behavioral dynamics in terms of intra-subjective variability in a reward-related task in individuals with SZ. As expected, SZ participants exhibited longer mean response times and lower accuracy in the discrimination task, as well as longer mean response times in liking ratings compared to HC—these results replicate recent findings. Extending previous findings, SZ exhibited abnormal behavioral dynamics, that is, enhanced intra-subject trial-to-trial variability across all four behavioral measures: response times in both the discrimination and liking tasks, as well as in accuracy and liking ratings. This increased intra-subjective variability appears to be somewhat task-unspecific, manifesting across different tasks (including different conditions within the reward task) and different behavioral measures such as RT, ACC, and liking ratings. That is further supported by the significant correlation of the intra-subject variabilities of the different behavioral measures across the distinct tasks.

Next, we observed a significant relationship of the enhanced intra-subject variability with both total and general psychopathological symptom severity. Notably, we found that the correlations between intra-subjective variability and symptom severity are significantly stronger than those between mean values and symptom severity. Collectively, our findings highlight abnormal behavioral dynamics in SZ with task-unspecific increases in intra-subjective variability which, unlike the measures of the mean performance, play a key role in psychopathological symptom severity. This suggests a basic temporal instability to be manifest in SZ not only in the cognitive domain (see introduction) but also in the affective domain of reward.

### Increased intra-subject variability—abnormal temporal dynamics in behavior

We observed abnormally long response times and decreased accuracy in our tasks in their respective mean values. This aligns well with recent research in SZ, suggesting our results converge with those of previous studies^22–24^. Our study lies in the observation of increased intra-subject variability in all behavioral measures, indicating higher trial-to-trial variability as measured by the CV. Unlike inter-subject variability, which highlights differences between subjects, intra-subject variability reflects the temporal changes over time within an individual, capturing the dynamic processes from trial to trial^4,10^. Our results clearly demonstrate enhanced dynamics as manifest in increased intra-subject variability that is not affected by the different means, given that we controlled for this by measuring CV^9,10^. The shorter timescale of high variability, referred to as inconsistency^5,6^, is thought to reflect fluctuations in cognitive processes involved in the continuous information processing^6,10,11^. In our study, increased intra-subject trial-to-trial variability was observed in all four behavioral measures and the different conditions. This suggests that increased intra-subject variability seems to manifest across different tasks as well as distinct domains, including affective-motivational (our results), sensory^14^, and cognitive^1,4^. Together with these results, our findings suggest abnormal temporal dynamics, e.g., variability and irregularity^14^, in SZ across different domains in a task-unspecific way at the psychological-behavioral level. Hence, temporal instability seems to underlie and modulate distinct functions including cognitive, sensory, and affective-motivational functions, suggesting that it may be a basic or fundamental disturbance in SZ.

### Abnormal temporal dynamics in behavior—relation to symptoms

The relevance of such abnormal temporal dynamics is further supported by our other findings with respect to the symptoms. Specifically, we show that intra-subject variability, as measured by CV, in our behavioral measures relates to symptom severity: the higher the intra-subject CV in behavioral measures such as RT and ACC, the more severe the total and general psychopathological symptoms. Noteworthy, we found such relationship only for the CV but not for the mean values. This suggests a special role for intra-subject variability with respect to the symptoms.

To understand that we need to clarify what exactly intra-subject variability measures. Intra-subject variability, e.g., CV, measures the temporal dynamics of cognitive processing from trial to trial^6,13,25^; that must be distinguished from the mean of the cognitive performance, which “averages out” the underlying trial-to-trial changes/dynamics when calculating the mean as average across all trials^4,6^. Indeed, previous research suggests that relying solely on mean measures of behavioral performance may overlook important cognitive differences among individuals and subtle behavioral variations, potentially leading to inaccurate inferences^4,6,7,10,26^. Accordingly, our findings that the PANSS correlates only with the CV but not the mean, suggest that symptom severity may be particularly related to the temporal dynamics underlying the cognition during our effective-motivational task (as measured by the intra-subject variability).

How could such abnormal temporal dynamics impact symptom severity? Enhanced intra-subject variability, reflecting abnormal temporal dynamics, suggests that the stimuli are processed in a temporally unstable way from trial to trial. In other words, the same stimulus is processed slightly differently across trials^25,27–30^. Previous studies reveal that SZ report temporal fragment of thought and time experience, reflecting the difficulties in perceive and integrate information in a continuous way^31,32^, which precented in delusions and disorganization of thought^32,33^. In addition, our data also show that such abnormal temporal dynamics extends to the affective-subjective component in our task, as reflected in the higher intra-subject variability of liking ratings. This finding aligns with previous literature, which indicates that reduced precision in the timing with increased temporal instability of conscious perception^32^. Such temporal instability may play a key role in disrupting a continuous sense of time^32^ which, in turn, may shape the various psychopathological symptoms in schizophrenia^31^.

### From abnormal temporal dynamics in behavior to the brain—spatiotemporal shaping of symptoms

Finally, one may want to raise the question of the sources of such abnormal temporal instability on the psychological-behavioral level. Increased intra-subject variability means that the temporal processing of each trial and its associated cognition and behavior is unstable and irregular from trial to trial, that is, each trial is processed in a slightly distinct temporal way compared to the other trials^20^.

Analogous instability and irregularity in the temporal features of the trials can be observed on the neural level. Recent EEG studies show that the temporal features of the amplitude during task paradigms varies strongly from trial to trial in SZ—this results in lower amplitude of the event-related potentials when averaged across the different trials within a task^20^. Enhanced amplitude fluctuations in SZ are also observed in their resting state’s moment-to-moment neural variability^11^. Similar observations of temporal instability have also been reported for the phase (as distinct from the amplitude), where its temporal features—such as phase coherence across trials, as measured by the intertrial phase coherence—show abnormally high variance and thus incoherence in its phase angles across different trials in both rest^24,34–37^ and task^21,28^ states.

This temporal imprecision in neural activity may reflect deficits in processing information in a temporally stable way over time, leading to impairments in behavioral performance^20,38^. Interestingly, this abnormal variability in the temporal features in SZ may occur across multiple timescales—not only at the millisecond level but also in moment-to-moment fluctuations as well as day-to-day or week-to-week changes^6,10,32,34,39^. While specifically at the second-timescale level, variability can also be observed in behavioral responses^6^, reflecting fluctuations in cognitive and affective-motivational processes^10,11^.

**Fig. 4:**
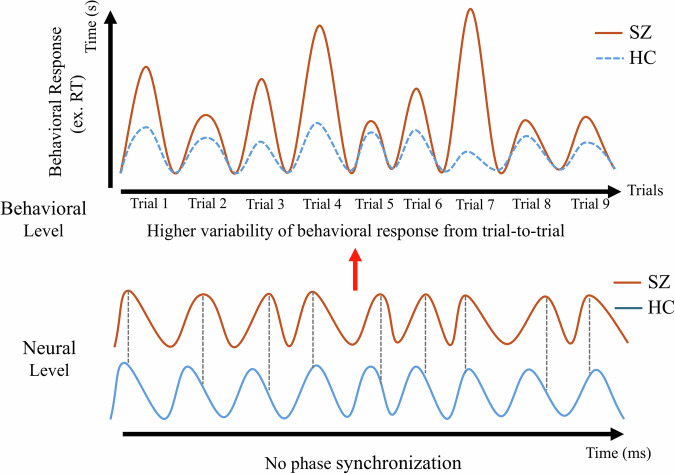
Temporal Imprecision at Neural and Behavioral Levels. In schizophrenia, temporal imprecision at the behavioral level, reflected by increased intra-subject variability, has been associated with temporal imprecision at the neural level. This imprecision in both the phase and amplitude of neural signals may indicate deficits in information processing, which in turn contribute to impaired behavioral performance. The figure illustrates the neural correlates of intra-trial variability in individuals with schizophrenia, highlighting the association between trial-to-trial variability in behavior and abnormal temporal dynamics in brain activity.

Taking together, we tentatively assume that the increased intra-subject variability in our behavioral measure, and consequently their temporal instability, may be related to a somewhat corresponding increased intra-subject variability and temporal instability at the neural level, particularly in the amplitude and phase of neural activity (Fig. 4). This temporal instability, reflecting temporal imprecision, may thus be shared by both neural and psychological-behavioral level as their “common currency”^40^. Given that temporal imprecision at both neural^20,34^ and psychological-behavioral^14,41,42^ levels relates to symptom severity, one may assume strong dynamic—this suggests spatiotemporal shaping of the schizophrenia symptoms as postulated in Spatiotemporal Psychopathology^43,44^.

In conclusion, we investigated the intra-subject variability in the behavioral measures of a reward-related task in SZ. Our findings show that SZ exhibits increased intra-subject variability across all four behavioral measures: response times in both discrimination task and liking, as well as accuracy and liking ratings. This reveals a task-unspecific increase in intra-subjective variability on the psychological level, suggesting abnormally elevated behavioral dynamics. Further, we observed a significant correlation elevated intra-subjective variability among the distinct behavioral measures themselves as well with and the severity of psychopathological symptoms.

In summary, our study demonstrates increased, task-unspecific intra-subjective variability in SZ. Such abnormal temporal dynamics in SZ at the psychological-behavioral level converge with analogous observations of temporal imprecision on the neural level. This provides further support for the framework that temporal imprecision is a ‘basic disturbance’ of schizophrenia^21,45^.

### Methodological limitations and future directions

The relationship between variability and symptoms in schizophrenia is not entirely consistent across studies^8^. While our results reveal a strong correlation between increased intra-subject behavioral variability and symptom severity, other research suggests that intra-subject variability may be more closely linked to cognitive performance rather than symptoms^2,46^. These inconsistencies could be due to differences in behavioral paradigms and symptom classifications^1,4,8^. Additionally, other studies indicate that inter-subject variability is related to cognition^47–49^. Alternatively, intra- and inter-subject variability might reflect partially distinct variables, whose relationship remains to be studied. For instance, one may raise the question of whether the observed increase in intra-subject variability accounts for the often-observed increase in inter-subject variability across various cognitive tasks^47–49^.

Extending this issue, variability can be calculated in different ways, such as intra-subject variability and inter-subject variability. Each type of variability has different indicators, like the CV, or parameters like μ, σ, and τ in the ex-Gaussian model. Intra-subject variability may reflect fluctuations within individuals across different tasks or conditions^18,25,50,51^, capturing the dynamics of how individuals respond to continuous stimuli. In contrast, inter-subject variability refers to differences between individuals. Therefore, studying the distinction and relationship between intra-subject variability and inter-subject variability, as well as their relationships with cognitive processes and symptoms in schizophrenia, is crucial for identifying potential biomarkers. Furthermore, the study did not include measures of IQ or cognitive tasks related to information processing speed, which may also be relevant to understanding abnormal intra-subject variability and its relationship to general symptoms of psychopathology. Future research could benefit from incorporating these measures to explore their potential contribution to the observed patterns in SZ.

Finally, our observation of differential effects of medication on the mean and CV remains unclear. While the medication has impact on the CV and thus the intra-subject variability of our behavioral measures, it did partially affect the mean of the same measures. Intra-subject variability reflects the temporal features and thus the dynamics of the behavioral measures during a cognitive task within the same subject^52^. In contrast, the mean relates more to the cognitive performance of the subject rather than its underlying dynamics. Therefore, the possibility of medication effects cannot be excluded^13,14^.

## Methods

### Participants

Participants included 90 patients diagnosed with schizophrenia and 28 healthy controls. Participants with schizophrenia were recruited from the outpatient clinics and day care center in the Department of Psychiatry, National Taiwan University Hospital. Diagnoses were based on the DSMIV-TR, administered by senior psychiatrists. Participants in the healthy control group were interviewed by trained assistants with the Chinese version of the Diagnostic Interview for Genetic Studies (DIGS)^53^, for screening out various psychiatric disorders. Exclusion criteria for all participants included (a) age above 50 years or below 20 years; (b) history of epilepsy or brain surgery; (c) diagnosis of bipolar disorder or schizoaffective disorder; (d) diagnosis of organic mental disorders, neurological disorder, or brain lesions; (e) pregnancy; or (f) diagnosis of substance abuse (except nicotine or caffeine) within the past 6 months. There were no differences between the schizophrenia and healthy control groups in age, gender, or education level. The demographic and clinical characteristics of the participants are presented in Table 1. The study was approved by the Research Ethics Committee of National Taiwan University Hospital. All participants provided written informed consent before the experiment started.

**Table 1:** Demographic characteristics of the sample.

	SZ (*n* = 90)	HC (*n* = 28)	Statistic	*p*
Age (years)	36.08 (7.59)	36.81 (7.65)	1149.50^a^	.48
Gender (M/F)	43/47	13/15	0.02^b^	.90
Education (years)	13.93 (2.79)	14.86 (2.19)	919.50^a^	.06
Onset	21.81 (5.33)	–	–	–
DOI (years)	14.15 (8.38)	–	–	–
CPZ (mg/day)	344.66 (253.94)	–	–	–
**PANSS Marder dimensions**				
Positive symptom	17.30 (5.91)	–	–	–
Negative symptom	16.39 (6.39)	–	–	–
Disorganized thought	13.37 (5.59)	–	–	–
UHE	5.13 (2.00)	–	–	–
AD	6.16 (2.31)	–	–	–
**PANSS scale**				
PANSS total	59.02 (16.51)	–	–	–
PANSS general	27.69 (8.10)	–	–	–
**SANS scale**				
SANS total	26.66 (17.41)	–	–	–
SANS global	8.58 (5.12)	–	–	–
SANS affective	9.31 (5.72)	–	–	–
SANS alogia	4.66 (4.04)	–	–	–
SANS avolition-apathy	4.58 (3.29)	–	–	–
SANS anhedonia-asociality	5.42 (4.02)	–	–	–
SANS attention	2.69 (2.37)	–	–	–

The values are presented as the mean (standard deviation).

*SZ* patients with schizophrenia, *HC* healthy controls, *DOI* duration of illness, *CPZ* chlorpromazine equivalent doze, *PANSS* positive and negative syndrome scale, *UHE* uncontrolled hostility/excitement, *AD* anxiety/depression.

^a^Mann–Whitney U tests were used to assess between-group differences.

^b^Sex distributions were compared using the chi-squared test.

### Clinical assessment

Symptom assessments included the Positive and Negative Syndrome Scale (PANSS) and the Scale for the Assessment of Negative Symptoms (SANS)^54,55^. Due to the heterogeneous nature of schizophrenia, a 5-factor model known as the Marder factors was developed to assess the range of clinically relevant schizophrenia symptoms^56^. This model, based on patient responses to PANSS items, has shown high internal consistency and reliability in both acute and stable patients^56–58^. The five factors include positive symptoms, negative symptoms, disorganized thought, uncontrolled hostility/excitement (UHE), and anxiety/depression (AD).

### Revised monetary incentive delay tasks

#### Procedure

**Fig. 5:**
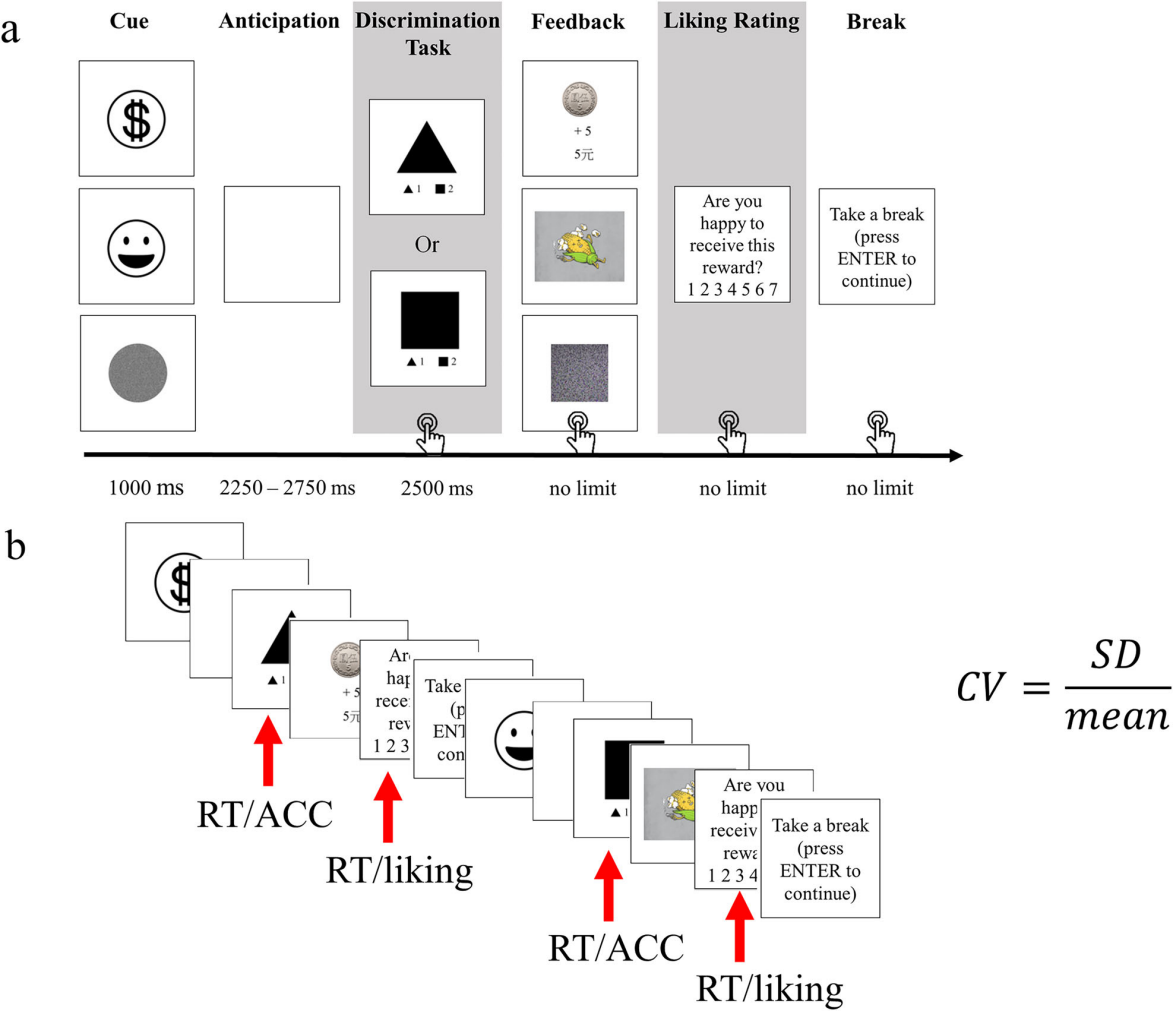
Behavioral paradigm of the revised monetary incentive delay task. **a** Behavioral paradigm of MID task. **b** Trial-to-trial variability is computed separately for RT, ACC, and liking ratings using CV. CV coefficient of variation, RT response time, ACC accuracy.

The study used an adapted monetary incentive delay (MID) task^15,59^, including three types of rewards: monetary, humor, and non-reward. The task consisted of four blocks with 24 trials each, yielding a total of 96 trials (32 monetary, 32 humor, 32 non-reward). During reward anticipation phase, a reward-related cue was presented in 1000 ms, followed by a delay period of 2250 to 2750 ms, after which participants need to complete a discrimination task. In the discrimination task, participants were asked to press a button in response to a target as quickly as possible in order to receive a reward. The screen displayed either a triangle or a rectangle: participants pressed ‘1’ for a triangle and ‘2’ for a rectangle. Response time longer than 2500 ms were considered incorrect. In the reward consumption phase, participants who succeeded in the discrimination task received the reward, while incorrect or slow responses resulted in no reward. The reward consumption stage was self-paced, with participants pressing a button once they understood the reward. Afterward, a 7-point liking scale (1 = unhappy to 7 = happy) appeared, and participants were instructed to rate how much they liked the reward they had just received. A short break followed each trial, and participants could press a button to begin the next trial when they were ready (Fig. 5a).

#### Statistical analyses

Data analyses were conducted with Jamovi (version 2.4.11) and R. For demographic characteristics, Mann–Whitney U and chi-square tests were applied, as tests of normality indicated that age and education were not normally distributed. To examine the variability in cognitive process and subjective experience, and their relationship to symptoms, we focused primarily on the discrimination task and liking ratings (Fig. 5b). Given the group difference in education at trend-level (*p* = 0.06), we controlled for education as a covariate in all relevant analyses. First, the intra-subject variability for each participant in each group was calculated using the coefficient of variation (CV). The CV is a standardized, unitless measures of variability that does not depend on the scale of the data^60^. It is traditionally defined as the ratio of the standard deviation to the mean. We measured response time (RT) and accuracy (ACC) in the discrimination task, as well as RT and ratings in the liking. The mean values for these four indicators were also analyzed, and the distributions of both the mean and CV values were provided. Since tests of normality showed that the mean RT of the discrimination task and the mean liking ratings were normally distributed, ANCOVA were used. For the other mean and CV values of the discrimination task and liking ratings, normality tests indicated non-normal distributions, so Quade’s test for non-parametric ANCOVA were applied. To examine interaction effects, a 2 (group) × 3 (reward types) mixed factorial ANOVA were used for the mean response time of the discrimination task. For all other non-normality indicators in both the discrimination task and liking ratings, 2 (group) x 3 (reward types) robust mixed factorial ANOVA was performed using the R package WRS2. Second, bivariate correlations were employed to investigate the relationships between variability and symptoms. Since the CV values from the discrimination task and liking ratings across all conditions were not normally distributed, partial Spearman correlations were applied. Given the large number of correlations computed, all correlation coefficients were estimated using a permutation test (n = 10,000) with the R package jmuOutlier^61^ to evaluate whether the correlation coefficient was significantly different from zero. For each permutation, the values of one data set were randomly reshuffled, and the number of times the absolute correlation value from the reshuffled data was greater than or equal to the original correlation was calculated. This count was then divided by the total number of permutations to compute the p-value. The statistical significance of the tests is reported using standard star notation (i.e., * indicates *p* < 0.05; ** indicates *p* < 0.01; *** indicates *p* < 0.001).

### Reporting Summary

Further information on research design is available in the Nature Research Reporting Summary linked to this article.

## Supplementary information

Supplementary_clean.data

## Data Availability

The data that support the findings of this study are available from the corresponding author (C.M.L.) upon reasonable request.
