# Supplementary material for: Increased intra-subject variability in reward behavior relates to symptom severity in schizophrenia
Source: Schizophrenia (Heidelb). 2025 Aug 5;11(1):108. doi: 10.1038/s41537-025-00645-7 (PMC12325774; doi:10.1038/s41537-025-00645-7)
Supplement: Supplementary file 1 — Supplementary_clean.data [file 41537_2025_645_MOESM1_ESM.docx]

**Increased Intra-subject Variability in Reward behavior Relates to Symptom Severity in Schizophrenia**

I-Fei Chen¹², Yu-Chen Chan³⁴*, Chih-Min Liu⁵*, Yi-Ting Lin⁵, Ming H. Hsieh⁵, Tzung-Jeng Hwang⁵, Tai-Li Chou⁶, Chen-Chung Liu⁵, Yi-Ling Chien⁵, Georg Northoff²*

¹ School of Psychology, University of Ottawa
² Mind, Brain Imaging and Neuroethics Unit, Institute of Mental Health Research, Royal Ottawa Mental Health Centre, University of Ottawa
³ Institute of Learning Sciences and Technologies, National Tsing Hua University, Hsinchu, Taiwan
⁴ Cognitive and Hedonic-Affective Neuroscience Laboratory, National Tsing Hua University, Hsinchu, Taiwan
⁵ Department of Psychiatry, College of Medicine and National Taiwan University Hospital, National Taiwan University, Taipei, Taiwan
⁶ Department of Psychology, National Taiwan University, Taipei, Taiwan

*Co-corresponding Authors:
**Yu-Chen Chan**
Address: No. 101, Sec. 2, Kuang-Fu Road, Hsinchu, 30013, Taiwan
Telephone: 886-3-5743043
Email: ycchan@mx.nthu.edu.tw

**Chih-Min Liu**
Address: No. 1, Changde St., Zhongzheng Dist., Taipei City, 100229, Taiwan
Telephone: 886-2-2312-3456 ext 66791
Fax: 886-2-2375-3663
Email: cmliu1968@ntu.edu.tw

**Georg Northoff**
Address: 1145 Carling Avenue, Ottawa, ON K1Z 7K4, Canada
Telephone: 613-722-6521 ext. 6959
Fax: 613-792-3935

Email: georg.Northoff@theroyal.ca

**Supplementary Tables**

|  | Mean RT^1^ | CV RT^2^ | Mean ACC^2^ | CV ACC^2^ |
| --- | --- | --- | --- | --- |
| PANSS total | 0.09 | 0.26* | -0.37*** | 0.36*** |
| PANSS general | 0.07 | 0.23* | -0.38*** | 0.37*** |
| SANS total | 0.04 | 0.13 | -0.27* | 0.27* |
| SANS global | 0.07 | 0.16 | -0.27* | 0.27* |

**Table S1. Correlation Between Clinical Scales and the Mean and CV of Response Time and Accuracy in a Discrimination Task** *Note:* RT = response time. ACC = accuracy. PANSS = positive and negative syndrome scale. SANS = scales for the assessment of negative symptoms. ^1^Partial Pearson's correlation analysis was used. ^2^Partial Spearman's correlation analysis was used. **P* < .05; ***P* < .01; ****P* < .001.

|  | Mean RT^1^ | CV RT^1^ | Mean liking ratings^2^ | CV liking ratings^1^ |
| --- | --- | --- | --- | --- |
| PANSS total | 0.17 | 0.05 | -0.17 | 0.22* |
| PANSS general | 0.15 | 0.01 | -0.15 | 0.22* |
| SANS total | 0.07 | -0.10 | -0.09 | 0.13 |
| SANS global | 0.12 | -0.05 | -0.06 | 0.12 |

**Table S2. Correlation Between Clinical Scales and the Mean and CV of Response Time and Liking Ratings** *Note:* RT = response time. PANSS = positive and negative syndrome scale. SANS = scales for the assessment of negative symptoms. ^1^Partial Spearman's correlation analysis was used. ^2^Partial Pearson's correlation analysis was used. **P* < .05; ***P* < .01; ****P* < .001.
